# IRSDT: A Framework for Infrared Small Target Tracking with Enhanced Detection

**DOI:** 10.3390/s23094240

**Published:** 2023-04-24

**Authors:** Jun Fan, Jingbiao Wei, Hai Huang, Dafeng Zhang, Ce Chen

**Affiliations:** Army Aviation Institute, Beijing 101121, China

**Keywords:** infrared image, object detection, target tracking, yolo, KCF

## Abstract

Currently, infrared small target detection and tracking under complex backgrounds remains challenging because of the low resolution of infrared images and the lack of shape and texture features in these small targets. This study proposes a framework for infrared vehicle small target detection and tracking, comprising three components: full-image object detection, cropped-image object detection and tracking, and object trajectory prediction. We designed a CNN-based real-time detection model with a high recall rate for the first component to detect potential object regions in the entire image. The KCF algorithm and the designed lightweight CNN-based target detection model, which parallelly lock on the target more precisely in the target potential area, were used in the second component. In the final component, we designed an optimized Kalman filter to estimate the target’s trajectory. We validated our method on a public dataset. The results show that the proposed real-time detection and tracking framework for infrared vehicle small targets could steadily track vehicle targets and adapt well in situations such as the temporary disappearance of targets and interference from other vehicles.

## 1. Introduction

Infrared signals, which have relatively low levels of atmosphere scattering, contain spontaneous emission information about the target. Thus, an excellent method for detecting and tracking long-distance thermal targets involves conducting research and analysis of the target characteristics of long-wave infrared video images. Currently, the long-wave infrared image-based small target detection method is widely used in fields such as forest fire prevention, field search and rescue, and night reconnaissance. In these tasks, the target object often presents grayscale features different from the background, making it easy to differentiate them. However, other factors, such as low resolution, high noise, and single-channel information of infrared images, have also made it more difficult to detect small targets. According to the analysis of the environment, on the one hand, some background objects in the wilderness scene have grayscale or shape information similar to that of the target. On the other hand, the target appears deformed, hidden, or invisible while moving. These factors make it difficult to detect and track small infrared targets in environments with complex backgrounds.

Target detection methods include traditional and convolutional neural network (CNN)-based methods. In traditional algorithms, complex background filters and adaptive filter coefficients must be manually designed. Compared with the traditional methods, the CNN-based detection model uses dataset samples to drive the parameter correction process, adaptively learning the common features and disregarding unnecessary features of the same type of target. Hence, the CNN-based detection model has a higher detection ability and robustness.

Currently, the CNN-based target detection method can be classified into Anchor-based methods and Anchor-free methods. The former includes one-stage methods, such as SSD [1] and the yolo series [2,3,4,5,6,7,8], and two-stage methods, such as R-CNN [9] and Faster RCNN [10]. The latter includes center-based methods, such as DenseBox [11] and FCOS [12], and keypoint-based methods, such as CornerNet [13] and ExtremeNet [14]. This study has primarily focused on using the one-stage method because it considers both high detection accuracy and fast detection speed regarding infrared small target detection tasks.

Researchers are currently providing many optimization proposals for enhancing the detection ability of the CNN-based infrared small target detection model. Several studies have analyzed the characteristics and motion states of infrared small targets and optimized the infrared small target detection algorithm in areas such as the spatial–temporal information [15,16,17], multiscale information [18,19,20,21], feature enhancement [22,23,24,25,26], and training and detection strategies [27,28], achieving relatively good detection results. However, most methods do not consider computing costs and parameter reduction in small models, limiting the likelihood of the CNN-based infrared small target detection model being used in edge computing hardware platforms.

Therefore, this study proposed a new infrared small target detection and tracking framework, comprising three stages: full-image target detection, cropped-image target detection and tracking, and target trajectory prediction. At the full-image target detection stage, we developed the yolo_IRS_1, a lightweight CNN-based infrared small target detection model that improves the recall rate of small vehicle targets without increasing computing costs. At the cropped-image target detection and tracking stage, we combined the CNN-based and traditional method. First, we developed the yolo_IRS_2, a mini network detection model for accurately detecting small targets. Next, we adopted a typical KCF tracker [29] to improve its adaptability to situations, such as target deformation and occlusions. At the target trajectory prediction stage, we developed a target trajectory predictor to improve the tracking stability in situations such as the target’s temporary disappearance and the interference of other similar objects. We verified this framework using publicly available infrared small vehicle target datasets [30], with the results demonstrating that the proposed real-time detection and tracking framework for infrared small vehicle targets can track target vehicles consistently while adapting relatively well to situations such as temporary disappearance of targets and interference from other vehicles.

The main contributions of this study can be summarized as follows:

1. We proposed a new infrared small target detection and tracking framework, which combines a CNN-based target detection model, a traditional target tracker, and a target trajectory predictor. The method has a relatively high reasoning speed and detection accuracy.
2. We propose a group of lightweight infrared small target detection methods-yolo_IRS_D1 and yolo_IRS_D2, which have similar inference speeds with the yolov5s but have higher accuracy. Yolo_IRS_D1 and yolo_IRS_D2 are used to detect the whole image and the region image, respectively, so the proposed framework can effectively reduce the computational cost by using different detection models.
3. We propose a target screening strategy that combines target detection, tracking, and trajectory prediction results so that the framework can achieve the tracking stability when the target is occluded by the background and disturbed by other objects.
4. The proposed method has been verified using publicly available infrared small vehicle target datasets. The results demonstrated that the proposed framework tracks the vehicle target consistently and adapts well to situations such as the temporary disappearance of the target and interference from other vehicles. The Euclidean distance of the coordinate deviation is ≤4 pixels.

## 2. Related Works

### 2.1. Infrared Small Target Detection Methods Basd on Deep Learning

The Spatial–temporal information: To improve the detection performance of small infrared pedestrian targets in the sequence, Park et al. [15] comprehensively considered the target’s time, spatial, and target shape information using the source, inter-frame residual, and background mask images as the input images in the detection model. Similarly, Yao et al. [16] used the infrared image sequence containing the target’s time and space information as input to the detection network to eliminate the interference of static noise points in the infrared image sequence to the infrared moving small target. Du et al. [17] proposed a target enhancement mechanism using inter-frame differences, IFEA, to target infrared small moving targets under a static background. This method calculates the difference between the current and previous frames and then superimposes it on the current image to conduct feature enhancement in the target area.

Multiscale information: Bai et al. [18] proposed a detection network with a cross- connected double-pyramid structure. This structure joins two opposite pyramid structures to fuse multi-scale feature map information, improving the model’s prediction of small object location. The downsampling layer will result in the loss of details of small targets; therefore, Ding et al. [19] proposed the SSD-ST as a solution. This method uses the shallow-layered high-resolution features as the output and suppresses the background information in the shallow-layer feature maps using adjacent deep-layer feature maps to be fused with the shallow-layer feature maps. Lan et al. [20] adopted the hourglass structured backbone network to fuse detailed and semantic information about the target. Considering the size of small infrared targets and the characteristics changes of the target in the network, the researchers introduced a semantic-level self-monitoring mechanism into the model so that the semantic information of the low-resolution layer and the detailed information obtained from the encoded information through the upsampling process is enough to guide the generation of better feature maps. Hou et al. [21] employ a similar strategy. They introduced fully connected layers in the hourglass-structured backbone network to improve nonlinear expression capabilities. In addition, the fully connected layer has a global receptive field, which helps to suppress background noise.

Feature enhancement: Considering infrared targets typically have a strong local contrast, Fan et al. [22] proposed an infrared small target detection framework using local grayscale enhancement and corner points detection. The potential target area is extracted using the gradient vector feature of the image target, which is then input into the CNN classifier, suppressing the complex background clutter in the non-target area. Ju et al. [23] proposed an image input filtering module with an hourglass structure. This module improved the response of the potential target areas while suppressing the background. However, such a module processes the original image directly without differentiating between background clutter and the target. Hou et al. [24] suggested the RISTD to replace image input with fused images. Hou et al. adopted parallel convolutional layers with different convolution kernel sizes, similar to RFBNet [25]’s concept, to extract the comparative information of small targets and their surroundings. Additionally, they designed a dual-branch parallel structure at the neck of the network, with one branch serving as the pooling downsampling branch to further obtain the target’s overall semantic information and the other as a branch to enable complete transmission of the small target information to the deep layers. Xi et al. [26] proposed a guided map module similar to a region spatial attention mechanism, which uses the characteristics of the infrared target’s high grayscale responses to locate and enhance the grayscale features of the potential areas of the infrared small targets.

Training and detection strategies: Bosquet et al. [27] proposed an STDnet, a video image detection method for small targets only. First, this method uses the RCN to detect the input image and extract the potential target area before cutting and stitching the potential area to create a small-scale high-resolution feature map. Finally, it will only precisely detect small-scale high-resolution feature maps. Studies have demonstrated that this method helps improve the detection precision and frame number of small targets while simultaneously reducing memory overhead. Based on this method and to avoid disturbance from different individuals of the same type, Bosquet et al. [28] introduced the spatial–temporal information in their method and proposed the STDNet-ST. This method uses the STDNet to simultaneously detect two adjacent frames in the sequence and conducts correlation calculations on the potential target areas in the adjacent frames to differentiate among different individuals of the same target type. Then, it combines pipeline filtering to limit the targets’ movement range of the frames before and after.

### 2.2. Infrared Small Target Tracking Methods

The research direction of the infrared small target tracking algorithm mainly includes correlation filter algorithms, particle filter algorithms, and so on.

Correlation filtering: Hou et al. [31] proposed a mean shift tracking algorithm based on improved similarity measurement. Firstly, the neighborhood background interference in the mean shift tracking algorithm for the small target is analyzed, and an improved Bhattacharyya coefficient similarity measurement method based on neighborhood background information fusion is proposed. Then, different weights are set according to the gray value of the target neighborhood to reduce the background interference and improve the robustness of small target tracking. Li et al. [32] proposed an infrared dim and small target tracking algorithm under complex background. They adopt an improved local binary pattern (LBP) scheme to represent the target texture feature and propose a joint gray texture histogram method for a more distinctive and effective target representation. The mean shift algorithm is used to accomplish target tracking.

Zhang et al. [33] used statistical methods to analyze the differences in grayscale and gradient characteristics between infrared dim small target and background noise. Moreover, he constructed a small target model based on kernel probability density. This paper discusses the critical problems of infrared dim and small target tracking tasks, such as target template extraction, tracking position determination, and target template update. Qian et al. [34] proposed a method based on the singular value decomposition (SVD) and the improved kernelized correlation filter (KCF). In this method, the background information of the infrared image is estimated by the SVD method, and the dim target is enhanced by subtracting the corresponding estimated background with the update from the original image. After that, the KCF algorithm is combined with gaussian curvature filter (GCF) to reduce noise and estimate the target position, respectively. The target position is estimated with a response map, which is obtained via the kernelized classifier.

Yun et al. [35] proposed a histogram method based on target temperature information instead of grayscale information to improve the robustness of the mean shift method. Xiao et al. [36] proposed an infrared small target tracking algorithm based on multi-feature fusion and Kalman filtering. The innovation of the method is to summarize the characteristics of the reference region through three dimensions of grayscale, neighborhood contrast, and target motion cues and then generate a confidence map to predict the location of the small target. The weights of the three dimensions are updated after each frame of tracking.

Zhou et al. [37] proposed an infrared dim small target tracking method based on inter-frame correlation and continuity of target features. This method proposed a region constraint to eliminate the noise in nonadjacent regions and a confidence threshold to distinguish the object from the neighborhood noise. In addition, the position prediction of the prior information and the pipeline filter improves the tracking stability when the target is occluded or temporarily disappears.

Particle filtering: Zhao et al. [38] proposed an infrared target tracking method that combines the Sparsity-based generative model (SGM) and guided filter-based discriminative classifier (GFDC) with the particle filter. In this method, the SGM model extracts an effective template histogram, which considers the spatial information of each image patch. The guided filter uses the structural information of the guidance image in order to smooth the input image while preserving the edges. The Bayesian classification is then applied to consider the background when the model is fused.

Huo et al. [39] proposed an improved resampling particle filter algorithm based on adaptive multi-feature fusion. They first establish an observation model based on the adaptive fusion of the features of the weighted grayscale intensity, edge information, and wavelet transform and then generate new particles based on residual resampling by combining the target position in the previous frame and the particles in the current frame with higher weights to improve the tracking accuracy and stability.

## 3. Proposed Method

### 3.1. Structure of the IRSDT

The infrared small target detection and tracking (IRSDT) framework proposed in this study includes a group of lightweight CNN-based target detection models, a traditional target tracker, and a target trajectory predictor.

**Figure 1:**
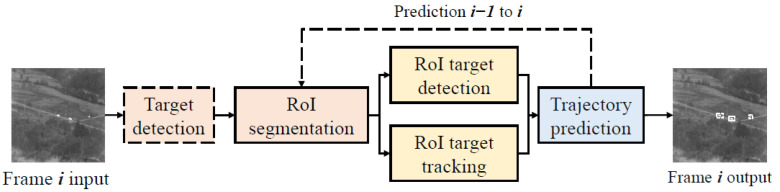
Proposed infrared small target detection and tracking framework.

The IRSDT consists of three stages, and its structure is shown in Figure 1. We proposed a CNN-based target detection model at the first stage to detect small targets in the entire image. The second stage had two parallel image processing branches, and we designed the yolo_IRS_2, a lightweight CNN-based target detection model in the first branch for the precise detection of small targets in the Ro. To improve the detection ability of deformed and partially occluded targets in the cropped image, KCF, a traditional target tracking algorithm, was included in the second branch. We designed a target trajectory predictor at the third stage to improve the tracking stability when the target disappeared temporarily and interference from other similar targets.

The yolo_IRS_1 did not process every frame during the inference of video images, because it has high computing costs.

### 3.2. Full-Image Target Detection

This section proposes a CNN-based infrared small target detection model, yolo_IRS_1. The model is used to detect infrared small targets in the full image.

**Figure 2:**
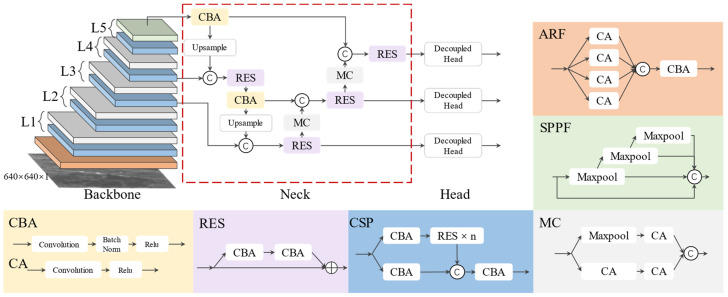
Structure of yolo_IRS_1.

Small infrared targets lack texture, details and are relatively small in size. Therefore, they are easily lost in the feature calculation process. Additionally, infrared images have a low signal-to-noise ratio owing to the performance of current infrared detection systems and environmental noise. There are several background objects with features similar to those of the target in the image, increasing the difficulty in differentiating between real and false targets. In this section, we propose yolo_IRS_1, a CNN-based target detection model (Figure 2), to improve the recall rate of infrared small targets.

Backbone: There are five layers in the backbone network of the yolo_IRS_1. Sequentially, L1-L4 contains CSP [6], a feature extraction network, and MC, a downsampling module. The CSP contains both a residual connection branch and a direct connection branch, which suppresses the loss of target features during information transfer. Each residual connection branch contains n RES modules with a bottleneck structure. Considering the size of the infrared small target and the computing costs of the CNN model, we set the n values of L3 and L4 to three; in the other layers, we set it to one. The MC layer is a downsampling layer comprising two parallel branches of the max pooling layer and strided convolutions. L5 contains a CSP layer and an SPPF layer in sequence, and its main purpose is to strengthen the semantic information of the target. We added the ARF [24], a self-adaptive module with four parallel branches, at the beginning of the backbone network. Each branch’s convolution kernel has a different dilated rate, making them receive region information of different sizes. This module has two advantages over large convolution kernels: first, it reduces computing costs while maintaining the size of the receptive field area almost constant. Second, it subsequently uses one convolution layer to adjust the weights of the various convolution branches to improve the adaptability to scale changes in the infrared target. In this study, the dilated rates of the four convolution kernels were 1, 2, 3, and 4, respectively.

Neck: We adopted the FPN + PAN structure on the neck of the network to promote the fusion of target semantics and details. We added a RES layer to each multi-scale fusion operation to reduce coupling among the different layers, smoothen gradient differences at different layers, and improve the effects of multi-scale fusion.

**Figure 3:**
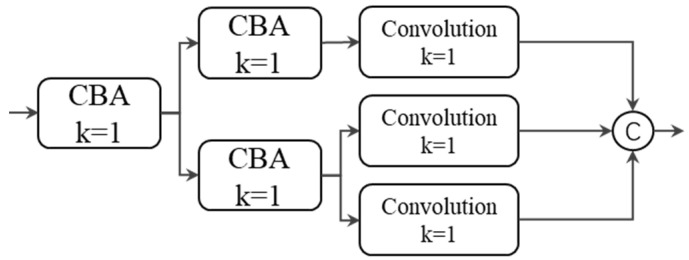
Decoupled head in yolo_IRS_1.

Detection head: We used a lightweight decoupling detection head [v6] for regression and classification, with its structure shown in Figure 3. The structure has two branches, one of which has channels equal to those of the target classification and is used to predict the target’s classification information. The other branch consists of two sub-branches for predicting the bounding boxes and confidence, respectively.

**Figure 4:**
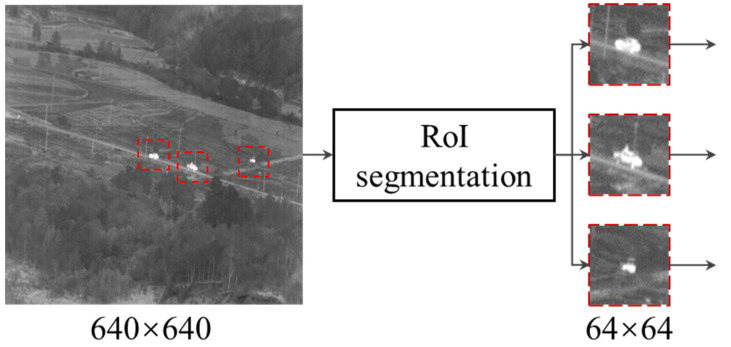
Examples of cropped images obtained by RoI segmentation.

After the full image target detection model has detected the target, we used the center of the target as the center of the region of interest (RoI) and crop out fixed-size images. We then used these cropped images in the next stages, according to the flow shown in Figure 4. The cropped image in this study has a size of 64 × 64 pixels. We chose the fixed-size segmentation size due to carefully considering the small target size distribution, the target inter-frame speed, and the computational overhead of the framework.

### 3.3. Cropped-Image Target Detection and Tracking

This section proposes an infrared small target detection and tracking method. The proposed method comprises a CNN-based lightweight infrared small target detection model, a traditional KCF tracker, and a novel target screening strategy.

After training, the CNN-based target detection model could find targets based on their characteristics; however, two problems remained. First, the CNN model was unable to fully learn the features of the target when the training sample did not contain sufficient description of the target. Second, the CNN model tended to classify the background features as the target features when the background in the training sample was relatively simple.

We added an RoI target tracking branch in parallel to the RoI target detection branch to address this challenge. This branch could extract the features of the target area in the current frame and compare those features for similarities against the image in the next frame to locate the target area’s best match. The target tracking algorithm had good adaptability to the deformation and occlusions of the target because it was not restricted by prior conditions. In this study, the selection of the tracking algorithm should consider its computational costs, tracking stability when the target is deformed and partially occluded. Therefore, we used a typical KCF algorithm as the RoI target-tracking algorithm.

The KCF method estimates the features of the target region through a multi-channel histogram of oriented gradients (HOG) information and maps it into a regression region with gray Gaussian distribution.

The formula is expressed as follows:

(1) g=h(f)

where *h*() is the filter, *g* is the regression region, and *f* is the features of the target.

The KCF method trains a filtering factor *h(z) = w^T^z* by ridge regression on kernel space, and the goal is to minimize the squared error between *f* and *g*.

(2) $$\underset{h}{\min}\overset{n}{\underset{i}{\sum }}{\left(g_{i}-f_{i}\right)}^{2}+\lambda {\left\|w\right\|}^{2}$$

The *λ* is a regularization parameter that controls overfitting, as in the SVM.

**Figure 5:**
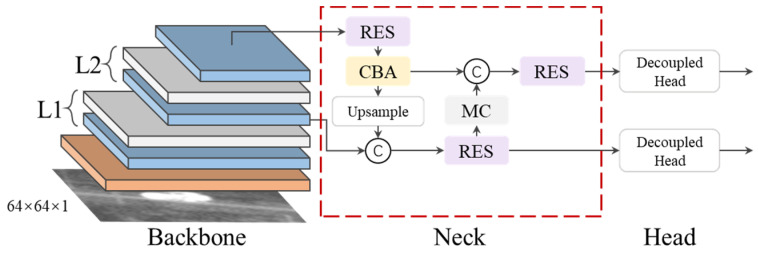
Structure of yolo_IRS_2.

In terms of RoI target detection, we designed yolo_IRS_2, a lightweight CNN-based target detection model, which structure is shown in Figure 5. The CSP layer, n, in L1 and L2, was set to three. The modules in yolo_IRS_2 were similar to those in yolo_IRS_1, but the former had lower computing costs than the latter. Its main function was to more accurately differentiate between the real and false targets and determine the target boundaries.

During the reasoning phase, the RoI image is simultaneously input into the RoI target detection model and the RoI target tracking algorithm; each branch estimates the target location separately. The output results can be divided into several situations based on the actual conditions, as shown in Table 1.

**Figure 6:**
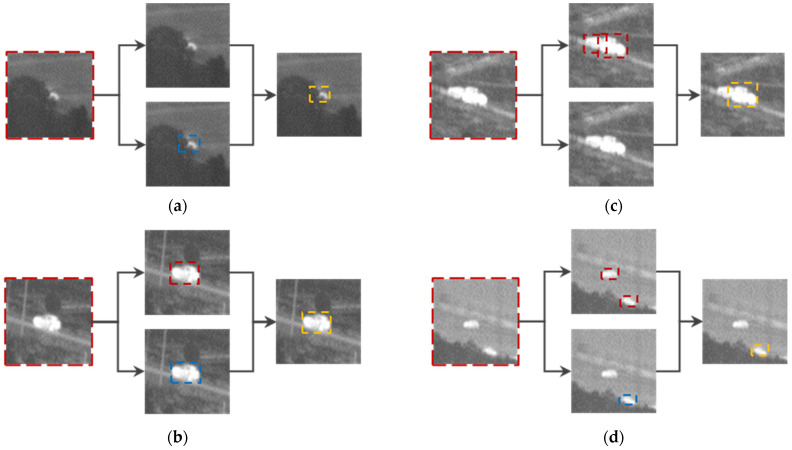
Example of RoI target detection and tracking reasoning results. The upper branch in each figure is the detect branch and the lower branch is a tracking branch. (**a**–**d**) correspond to situations 2, 3, 4, and 5 in Table 1, respectively. The detect, tracking, and final results are highlighted in red, blue, and yellow, respectively.

The RoI target detection and tracking results are shown in Figure 6.

### 3.4. Target Trajectory Predictor

In this section, we propose an inter-frame object prediction method so the framework can still estimate the object’s coordinates when the detection and tracking methods cannot find the object.

As the CNN-based target detection model and target tracking algorithm were used to process single-frame images, evaluating the target’s motion characteristics was challenging. Therefore, the algorithm’s tracking accuracy was reduced when multiple targets were met and when the target temporarily disappeared from the image sequence.

To address this problem, we designed an inter-frame target trajectory forecast algorithm to estimate the change of pixel coordinates in the image. The algorithm is expressed as follows:

(3) $${\hat{x}}_{i+1}=\beta \cdot Kalman\left(x_{i}\right)+\left(1-\beta \right)\cdot MAa\left(x_{i}\right)$$

(4) $$MAa\left(x_{i}\right)=\left(x_{i}+v_{i}+\frac{1}{2}{\hat{a}}_{i}{}^{2}\right)$$

(5) $$v_{i}=x_{i}-x_{i-1}$$

(6) $${\hat{a}}_{i}=\frac{v_{i}-v_{i-1}+{\hat{a}}_{i-1}}{2}$$

(7) $$\beta =\{\begin{array}{c} \frac{k}{times}\;\;\;if\;\;\;k<times\\ 1\;\;\;\;\;\;\;\;\;\;\;\;\;k\geq times \end{array}$$

where ${\hat{x}}_{i+1}$ represents the pixel coordinate prediction value of the target in frame *i+1*. *x_i_* denotes the coordinates of the target in the image of the frame *i*, and *Kalman*() represents a typical Karman filtering algorithm. We designed a moving average acceleration (*MAa*()) method to replace the Karman filtering method because of a significant prediction error in the Karman filter algorithm during the early stages of operating the system. The *MAa*() function uses the moving average method to predict the acceleration of the target and its position in the next frame. *v_i_* represents the target’s relative velocity in frame *i*. ${\hat{a}}_{i}$ represents the predicted value of the relative acceleration of the target in frame *i*.

To smoothen the prediction value ${\hat{x}}_{i+1}$ during the handover process of the two methods, we designed a warm-up mechanism, where *β* and *times* were the warm-up factor and cycle, respectively.

**Figure 7:**
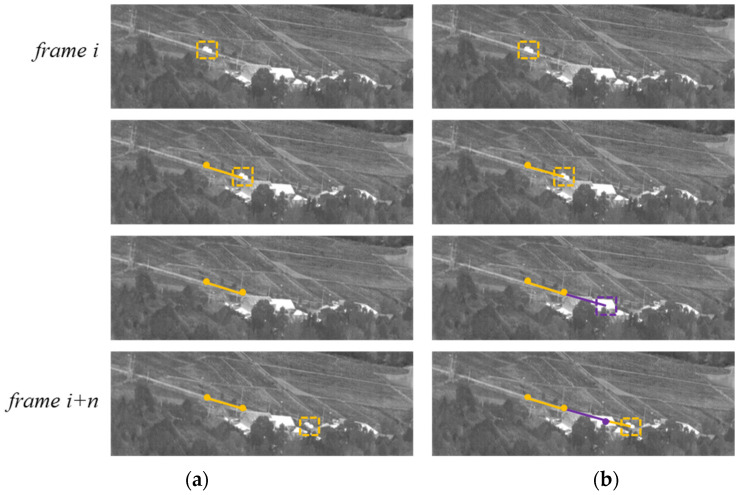
Example of target trajectory prediction results. (**a**) without trajectory prediction; (**b**) with trajectory prediction. The trajectory prediction algorithm could estimate the target location when the vehicle was obscured by a building. The prediction box and trajectory are labeled in purple.

The results of the target trajectory prediction results are shown in Figure 7.

## 4. Experiment Settings

### 4.1. Experiment Environment

The system platform used in this experiment was Ubuntu 20.04; PyTorch1.8.1 was used as the CNN model’s training framework, RTX3080Ti was used as the training platform, with the training cycle set to 50 epochs.

### 4.2. Dataset

**Figure 8:**
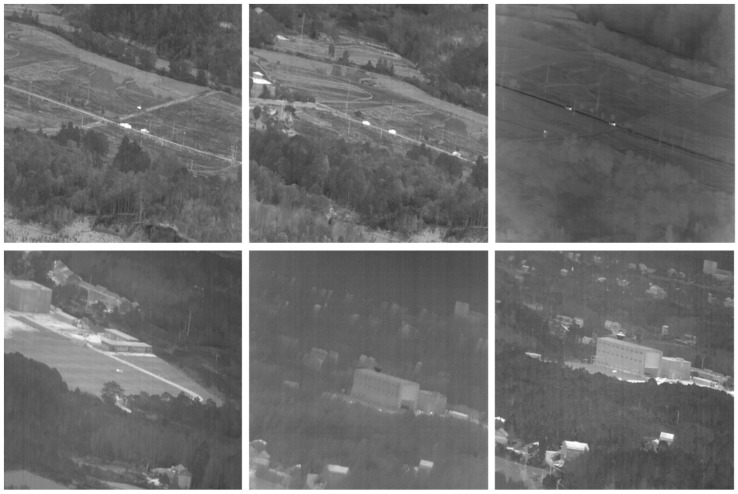
Some images from the dataset containing small objects with complex backgrounds.

The dataset used in this study consisted of infrared small target vehicles under a complex background [30]. Figure 8 shows some of the dataset’s images.

**Figure 9:**
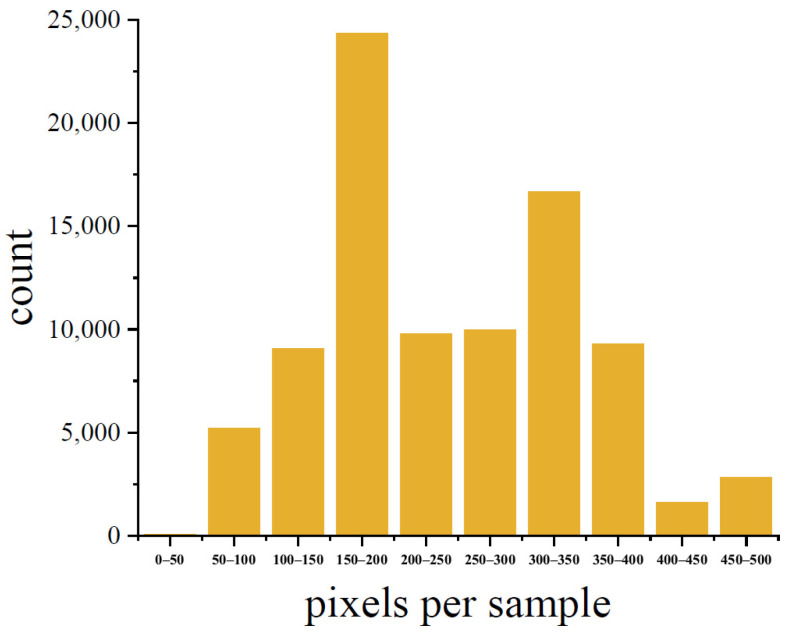
Vehicle dataset sample size distribution.

The dataset contained 21,750 images and 89,174 targets. These images consisted of 87 sets of image sequences and 393 trajectories. For the study of subsequent detection and tracking algorithms, the image resolution in the dataset was stretched to 640 × 640 pixels; the images were used as training samples for subsequent network models. Figure 9 depicts the size distribution of the stretched target.

To ensure the randomness and coverage of the training set, the odd-numbered group with 87 sets of images was used as the training set, totaling 11,000 images. The even-numbered group was used as a test set, which had 10,750 images. The distribution of the dataset is shown in Table 2.

To train and test the yolo_IRS_2, we cropped the images from the above dataset and created the RoI dataset. The resolution of the images in the RoI dataset was 64 × 64 pixels, and each image was the central area of the image in the infrared vehicle dataset.

The RoI data sequence distribution, with 8884 training and 9333 testing samples, was consistent with that of Table 2.

### 4.3. Evaluation Criteria

Target detection and tracking became more challenging because of the target’s texture and outline in the infrared small target image being obscured by features similar to those of some background objects. Therefore, during the experiment, we were more concerned with the interference from false targets to the CNN-based detection model.

We used the precision and recall rates frequently used for target detection tasks and the score calculation method recommended by the dataset in this study as evaluation criteria for the CNN-based detection model in this study. The precision rate, recall rate, and score are calculated as follows:

(8) $$Precision=\frac{TP}{TP+FP}$$

(9) $$Recall=\frac{TP}{TP+FN}$$

(10) Score=TP−FN−2·FP

where *TP* stands for true positive, *FP* for false positive, and *FN* for false negative.

For small targets with only a few pixels in width and height, the slight deviation in size and position between the predicted boxes and the ground truth will cause drastic changes in its IOU value. Hence, small infrared targets’ detection and tracking results are inaccurate using the IOU value. This paper uses the Euclidean distance between the center point of the prediction box and the label box’s center point as the evaluation criterion of the proposed framework. The evaluation formula is as follows:

(11) $$Euclidean\;distance=\left|x_{p}-x_{t}\right|+\left|y_{p}-y_{t}\right|$$

where (*x_p_*,*y_p_*) is the center coordinate of the prediction box, (*x_t_*,*y_t_*) is the center coordinate of the ground truth;

## 5. Experiment and Result Analysis

### 5.1. Ablation Experiment

We designed ablation experiments to evaluate the capabilities of our proposed optimized module for infrared small object detection. We modified the backbone, the neck of the network, and the detection head of the Yolov5s. Table 3 displays the experimental results based on a resolution of 640 × 640 pixels for the training and inference images.

According to Table 3, No. 1 is the Yolov5s model, and No. 4 is the yolo_IRS_1 model proposed in this study. “√” denotes replacing the Yolov5s module with the module proposed in this study. When comparing the results of No. 1 and No. 2, there were significant improvements in the precision and recall rates of the latter, particularly in the 66% decrease in false alarms, indicating that the designed backbone network strengthened the features of small targets and enabled the CNN model to more accurately distinguish between false and real targets. When comparing the results of No. 2 and No. 3, the proposed neck network reduced the number of missed detections decreased by 12.9%, indicating that the structure strengthened the differences between weak target features and the background through multi-scale information fusion, improving the CNN model’s detection effect on weak targets. Comparing the results of No.3 and No.4, the model that used the decoupling detection head significantly reduced the number of missed detections by 7.4%, whereas the number of false alarms slightly decreased, indicating that the decoupling detection head reduced the mutual interference between the classification and regression branches in the weak infrared target detection task while improving the capture ability of special targets. However, the separate prediction of the target classification and location also makes it difficult for the model to comprehensively evaluate the differences between the target and its background from various angles, thus resulting in increased false alarms.

### 5.2. Comparison of Advanced Detection Models

In this section, we compare the proposed model with the advanced one-stage infrared small target detection algorithm in recent years, and the results are shown in Table 4 below. Methods No. 2–No. 5 in the following table take yolov5s as the baseline. The methods from No. 6 to No. 9 take SSD as the baseline. In order to keep the computational overhead of the algorithms in the following table in the same order of magnitude, we set the width-multiple factor to 0.5 in the methods from No. 6 to No. 9.

The experimental results show that our model has an optimal recall rate and score, suboptimal precision rate and computational costs. It is proved that the proposed method performs well on the infrared small target detection task.

### 5.3. RoI target Detection Experiment

Table 5 compares Yolov5s with yolo_IRS_2. The training and inference image datasets were adopted from the RoI dataset. According to the experimental results, the CNN infrared small target detection model proposed in this study has higher precision and recall rates, increasing by 0.6% and 2.1%, respectively.

### 5.4. IRSDT Detection and Tracking Experiment

In this section, we used the No. 14 sequence and No. 80 sequence from the test set as the experimental objects, with each sequence comprising 250-image frames taken continuously under fixed camera conditions. The image scenes consisted of moving vehicles, forests, plains, buildings, and roads.

#### 5.4.1. Sequence No. 14

**Figure 10:**
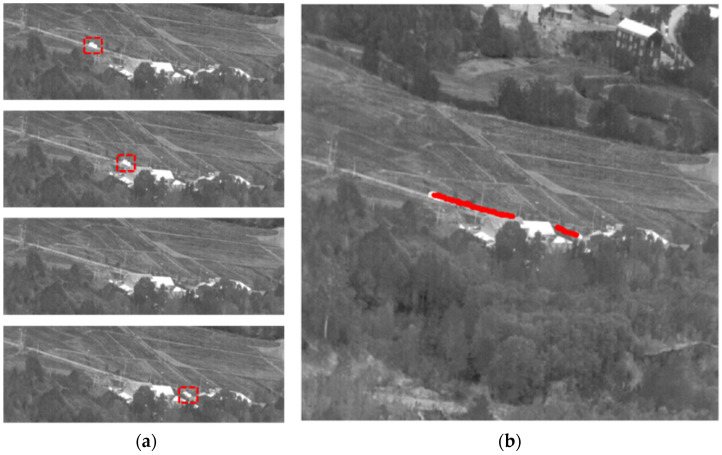
Sequence No. 14 (**a**) Target locations at different moments and (**b**) Target label trajectories throughout the sequence.

In sequence No. 14, the vehicle target was driving along the road from left to right, disappearing completely behind the building in frame 130 before reappearing in frame 200. Figure 10 shows the target image and label trajectories. The dataset did not have labels for targets when they disappeared behind buildings.

**Figure 11:**
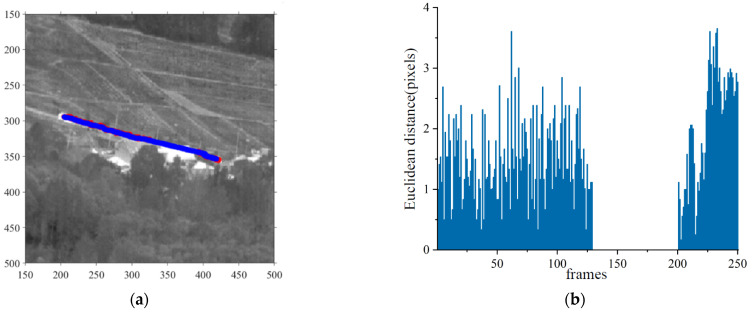
IRSDT detection and tracking results of vehicle targets. (**a**) shows the trajectory of the label and the inference in the real image; (**b**) shows the Euclidean distance between the inference and label coordinates of each frame in the sequence. The target had disappeared behind the building between frames 130 and 200, and the dataset had no label for the target; therefore, no pixel bias was calculated.

Inference was performed on image sequences using the proposed detection and tracking framework. Figure 11 shows the experimental results. The IRSDT could correctly identify the vehicle target and consistently track the target motions. When the object disappeared, the proposed method could predict the location of the target. According to Figure 11b, the tracking deviation of the target using the proposed algorithm was essentially within 2 pixels. During the second stage, the tracking deviation increased slightly to approximately 3 pixels when the target reappeared. This result demonstrates the effectiveness of the proposed framework in detecting and tracking infrared moving small targets.

#### 5.4.2. Sequence No. 80

**Figure 12:**
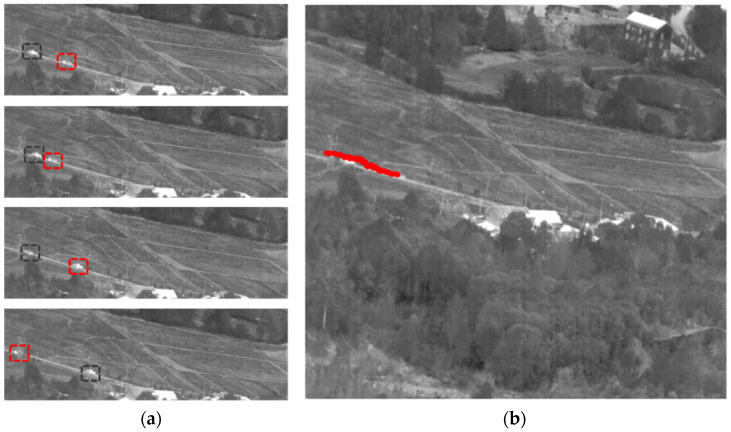
Sequence No. 80 of (**a**) target locations at different moments and (**b**) target label trajectories throughout the sequence. Vehicles requiring tracking were framed with red boxes, with the other vehicles framed with black boxes.

In Sequence No. 80, the vehicle target that required tracking was on the right side, driving from right to left. There was another vehicle along the path, and the two vehicles met at frame 115, while the target was behind the another vehicle. The intersection process ended at frame 160. The target completely disappeared behind the trees at frame 236 and reappeared at frame 243. Figure 12 shows the target image and label trajectories. The dataset did not have labels for targets when they disappeared behind the trees.

**Figure 13:**
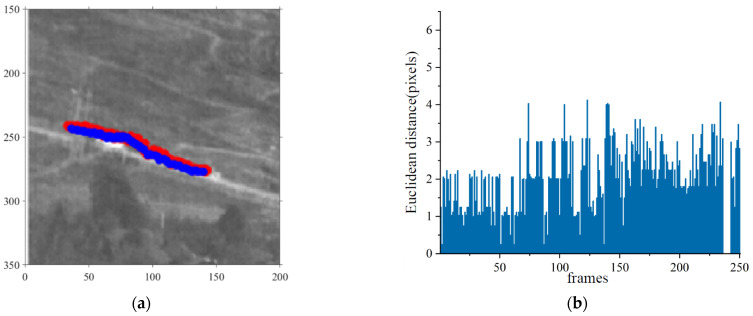
Detection and tracking results of vehicle targets using the proposed framework. (**a**) The trajectory of the label and the inference in the real image, and (**b**) the Euclidean distance between the inference and label coordinates of each frame in the sequence. The target disappeared behind the trees between frames 236 and 243, and the dataset had no label for the target; therefore, no pixel bias was calculated.

Inference was performed on image sequences using the proposed detection and tracking framework. Figure 13 shows the experimental results. The IRSDT could correctly detect the vehicle target and consistently track the target during the motion. The tracking deviation was approximately four pixels throughout the sequence. The proposed method could still identify a particular car for tracking when the target intersected with other vehicles with similar features.

## 6. Conclusions

In this study, we proposed a real-time detection and tracking framework for infrared small targets under complex backgrounds. First, we designed a CNN small target detection model to detect small targets in large-resolution images, targeting infrared small target vehicles’ features and background information. Second, we designed a lightweight CNN detection model and adopted a traditional KCF target detection algorithm for a more accurate evaluation of the location and size information of small targets in cropped images. Finally, we proposed a target trajectory predictor based on the optimized Kalman filtering method. The proposed method was verified using publicly available infrared small vehicle target datasets. According to the experimental results, the experimental results show that the proposed method in this study performed better than the other advanced small-object detection methods. The proposed infrared small target detection and tracking framework could accurately identify and track the target while performing well under interference from other similar targets and temporary target disappearances. The Euclidean distance of the coordinate deviation is ≤4 pixels.

## Figures and Tables

**Table 1:** RoI target detection and tracking results, with result screening.

S/N	RoI Target Detection	RoI Target Tracking	Final Results
1	No target	No target	Output RoI image center coordinates;
2	No target	With target	Output tracking results;
3	Single target	With and without target	Output detection results;
4	Multiple targets	No target	Output of detection results nearest to the center of RoI image;
5	Multiple targets	With target	Output of detection results nearest to the tracking result;

**Table 2:** Dataset sample distribution.

Class	Training Set	Test Set
Data Serial Number	1; 3; 5; 7; 9; 11; 13; 15; 17; 19; 21; 23; 25; 27; 29; 31; 33; 35; 37; 39; 41; 43; 45; 47; 49; 51; 53; 55; 57; 59; 61; 63; 65; 67; 69; 71; 73; 75; 77; 79; 81; 83; 85; 87; 89; 91	2; 4; 6; 8; 10; 12; 14; 16; 18; 20; 22; 24; 26; 28; 30; 32; 34; 36; 38; 40; 42; 44; 46; 48; 50; 52; 54; 56; 58; 60; 62; 64; 66; 68; 70; 72; 74; 76; 78; 80; 82; 84; 86
Number of Images	11,000	10,750
Number of Sample	44,131	45,043

**Table 3:** Comparison of results of detection models with different configurations.

No.	Backbone	Neck	Head	TP	FN	FP	Precision (%)	Recall (%)	Score
1				36,395	8648	3468	91.3	80.8	20,810
2	√			39,458	5585	**1178**	**97.1**	87.6	31,515
3	√	√		40,178	4865	1328	96.8	89.2	32,657
4	√	√	√	**40,539**	**4504**	1383	96.7	**90.0**	**33,268**

Bold number: optimal result.

**Table 4:** Comparison of different detection methods.

No.	Mdoels	TP	FN	FP	Precision (%)	Recall (%)	Score	MParam	Gflops	FPS
1	Yolov5s	36,395	8648	3468	91.3	80.8	20,810	7.06	**16.3**	**276**
2	Yolo-DGS [40]	37,791	7252	2584	93.6	83.9	25,371	9.4	64.3	154
3	IYolo [41]	38,242	6801	1760	95.6	84.9	27,920	3.5	34.2	109
4	Yolo-SASE [42]	39,683	5360	1870	95.5	88.1	30,583	13.7	28	262
5	ECA-Yolo [43]	39,998	5045	1885	95.5	88.8	31,184	11.3	23.1	256
6	FD-SSD [44]	29,683	15,360	1013	96.7	65.9	12,298	5.8	30.1	88
7	DF-SSD [45]	29,404	15,639	1007	96.7	65.3	11,752	10.0	31.6	192
8	SSD-ST [19]	33,512	11,531	**895**	**97.4**	74.4	20,192	**2** **.8**	24.8	244
9	FA-SSD [46]	31,620	13,423	1699	94.9	70.2	14,799	9.0	34.8	173
10	IRSDet (ours)	**40,539**	**4504**	1383	96.7	**90.0**	**33,268**	7.7	19.8	219

Bold number: optimal result and underline number: suboptimal result.

**Table 5:** RoI image detection results.

Model	Resolution	Target Number	TP	FN	FP	Precision (%)	Recall (%)	Score
Yolov5s	64 × 64	9333	8437	896	529	94.1	90.4	6483
yolo_IRS_2			8633	700	483	94.7	92.5	6947

## Data Availability

Datasets Link: http://www.doi.org/10.11922/sciencedb.j00001.00331.
